# Biocomplexity and Fractality in the Search of Biomarkers of Aging and Pathology: Focus on Mitochondrial DNA and Alzheimer’s Disease

**DOI:** 10.14336/AD.2016.0629

**Published:** 2017-02-01

**Authors:** Annamaria Zaia, Pierluigi Maponi, Giuseppina Di Stefano, Tiziana Casoli

**Affiliations:** ^1^Laboratory of Bioinformatics, Bioengineering and Domotics, Italian National Research Center on Aging - INRCA, via Birarelli 8, 60121 Ancona, Italy; ^2^School of Science and Technology, University of Camerino, via Madonna delle Carceri 9, 62032 Camerino (MC), Italy; ^3^Research, Innovation and Technology Transfer Office, Italian National Research Center on Aging - INRCA, via Birarelli 8, 60121 Ancona, Italy; ^4^Scientific and Technological Area, Italian National Research Center on Aging - INRCA, via Birarelli 8, 60121 Ancona, Italy.

**Keywords:** Aging, Alzheimer’s disease, Biocomplexity, Chaos Game Representation, Fractal lacunarity, mtDNA

## Abstract

Alzheimer’s disease (AD) represents one major health concern for our growing elderly population. It accounts for increasing impairment of cognitive capacity followed by loss of executive function in late stage. AD pathogenesis is multifaceted and difficult to pinpoint, and understanding AD etiology will be critical to effectively diagnose and treat the disease. An interesting hypothesis concerning AD development postulates a cause-effect relationship between accumulation of mitochondrial DNA (mtDNA) mutations and neurodegenerative changes associated with this pathology. Here we propose a computerized method for an easy and fast mtDNA mutations-based characterization of AD. The method has been built taking into account the complexity of living being and fractal properties of many anatomic and physiologic structures, including mtDNA. Dealing with mtDNA mutations as gaps in the nucleotide sequence, fractal lacunarity appears a suitable tool to differentiate between aging and AD. Therefore, Chaos Game Representation method has been used to display DNA fractal properties after adapting the algorithm to visualize also heteroplasmic mutations. Parameter β from our fractal lacunarity method, based on hyperbola model function, has been measured to quantitatively characterize AD on the basis of mtDNA mutations. Results from this pilot study to develop the method show that fractal lacunarity parameter β of mtDNA is statistically different in AD patients when compared to age-matched controls. Fractal lacunarity analysis represents a useful tool to analyze mtDNA mutations. Lacunarity parameter β is able to characterize individual mutation profile of mitochondrial genome and appears a promising index to discriminate between AD and aging.

The introduction in gerontologic field of paradigms, such as theory of complexity, chaos, and fractals, provide new tools and suggest new approaches to the study of aging processes. Apparent contradictions, or phenomena apparently unexplainable, become understandable in the light of these new paradigms. As an example, the variability observed in senescent phenotype can be explained in the light of the theory of complexity by considering longevity as a “secondary product” of evolution of a dynamic nonlinear system [1,2].

Complex systems are highly dependent upon initial conditions; thus, in a cohort, even very small differences occurring at certain times can cause higher and higher differences at later ages in most characteristics of individual phenotype. In this view, by considering living systems as complex systems [3], life trajectories of subjects in a population, whatever close at birth, will evolve fluctuating with time, progressively enlarging the variance of their phenotype characteristics, among which aging. Even in the presence of genetically homogeneous backgrounds, inter-individual variability is always present independently of how large environmental changes are. In fact, genetic-environment interaction causes unpredictable behavior at bifurcations (critical points at which changes of trajectory can occur). Bifurcations represent a source of variability responsible of heterogeneity of aging phenotype.

The concept that a complex system with a chaotic behavior often generates fractal structures, the so-called strange attractors [4], that can be observed at critical points, highlights the usefulness of fractal analysis as a suitable tool to measure biocomplexity and its changes with aging at both functional and structural levels [5-8]. Fractal analysis can measure variations of complexity in biosystems evolving with time by following different trajectories. Individual specific genetic-environment interactions define the senescent phenotype as normal aging, pathologic aging, or successful aging [2]. Fractal analysis, therefore, represents a promising tool to give insight into the search of good biomarkers useful to discriminate between physiologic and pathologic aging as well as between age-related and age-associated diseases, two main tasks dealing with aging well.

Alzheimer’s disease (AD) represents one major health concern for our growing elderly population. It accounts for increasing impairment of cognitive capacity followed by loss of executive function in late stage [9,10]. The expected increase in AD incidence [11] claims for a better understanding of the underlying etiology to achieve early diagnosis and effective treatments. However, AD pathogenesis is multifaceted and difficult to recognize. While a number of hypotheses have been proposed, the exact cause of AD is unknown. The most widely accepted hypothesis is the amyloid cascade hypothesis [12]: it posits amyloid-β (Aβ) plays an early and vital role in AD, as it triggers a cascade of events responsible for synaptic dysfunction, tau pathology, and neural loss [13].

An interesting hypothesis concerning AD development postulates a cause-effect relationship between accumulation of mitochondrial DNA (mtDNA) mutations and neurodegenerative changes associated with this pathology such as defective oxidative phosphorylation, increased oxidative stress, accumulation of Aβ, and apoptosis [14,15]. Human cells contain thousands of mtDNA copies and the most common mutations are insertions, deletions, and point mutations that are classified as homoplasmic or heteroplasmic if they regard all mtDNA copies or only a fraction of them, respectively [16,17].

When we approach studies on DNA sequences as it is searching for mutations in mtDNA, we have to face the problem of handling a huge data set by quite time consuming procedures. In addition, the wide variety of DNA mutations, in this particular context the mtDNA ones, found in both AD patients and control subjects makes the genetic characterization of AD very demanding during all kinds of analytical procedures used, both numerical and statistical.

Here we propose a computerized method for an easy and fast mtDNA mutations-based characterization of AD. The method has been built taking into account the complexity of living being and fractal properties of many anatomic and physiologic structures [18-21], among which is mtDNA [22]. The advent of fractal mathematics to describe complex structures in biology and medicine has been accepted by most scientists and an always increasing number of fractal analysis techniques have been proposed and applied. However, the application of fractal techniques is often limited to fractal dimension (DF). As a matter of fact, DF has been proposed and used as a suitable tool to measure complexity variation of most biomedical functions and structures during aging and disease [23-29]. This has been happening also in the case of DNA structure [30-34]. While DF gives an estimate of the complexity of the structure, it alone is not sufficient to characterize a fractal object. Lacunarity instead, another fractal property, describes the texture of a fractal and can measure fractal space filling capacity [35]. The term lacunarity, from Latin lacuna, lack or hole, was coined by Mandelbrot referring to gap distribution in a fractal [20,35], and lacunarity analysis was initially introduced to differentiate fractal objects displaying the same DF but having a very different appearance. A third fractal property, namely succolarity, introduced by Mandelbrot [36] has been recently reconsidered for analytical applications to further characterize fractal objects accounting for the connectivity of their structure [34,37,38].

Taking into account the limits of fractal analysis applied to natural objects [2,35,39], fractal lacunarity analysis has been used to develop our method. In fact, self-similarity and invariance of scale are properties belonging to ideal fractals: they show exact or approximate self-similarity. When fractal properties are considered in biology and medicine we deal with statistical self-similarity in a limited range of scale. Furthermore, multifractal structures are common. More recently, lacunarity analysis has been introduced as a more general technique able to describe random and fractal spatial patterns. Thus, lacunarity analysis can be used to describe the texture of complex objects with fractal, multifractal or non-fractal properties [39,40]. In addition, dealing with mtDNA mutations as gaps in the nucleotidic sequence, fractal lacunarity appears a suitable tool to differentiate between aging and AD. Therefore, in the present paper we provide a preliminary study where fractal lacunarity is candidate as a potential pathologic index regarding mutated mtDNA structure.

Chaos Game Representation method has been used to display DNA fractal properties [41-44] after adapting the original algorithm to visualize heteroplasmic mutations peculiar to mtDNA. Parameter *β*, from our fractal lacunarity method based on hyperbola model function [45-48], is the measure used to quantitatively characterize AD on the basis of mtDNA mutations.

## MATERIALS AND METHODS

### Subjects

This study was performed on 28 subjects, 14 AD patients and 14 age-matched controls, enrolled from October 2009 to September 2010 at the INRCA Geriatrics Unit of Fermo after approval by the Institutional Ethical Committee. Each subject included in the study, or caregiver when necessary, provided informed consent to participate. Patients were diagnosed according to National Institute of Neurological and Communicative Diseases and Stroke/Alzheimer’s Disease and Related Disorders Association (NINCDS-ADRDA) criteria for probable AD by an extended neuropsychological and functional evaluation, neuroimaging, and laboratory tests. Controls were volunteers and relatives of the patients and underwent the same diagnostic assessment as the AD group (Table 1).

**Table 1:** Characteristics of subjects included in the study

	AD patients	Controls	*p* Value
Number (M/F)	2/12	1/13	
Age	75.4 ± 5.1	73.1 ± 5.1	0.117
MMSE	17.3 ± 3.4	28.2 ± 0.8	<0.001
ADL	5.2 ± 1.3	6.0 ± 0.0	0.022
IADL	2.0 ± 1.4	8.0 ± 0.0	<0.001

Values are expressed as mean ± SD;

*p* Values have been calculated by one-tail t-test;

AD: Alzheimer’s Disease; MMSE: Mini Mental State Examination; ADL:

Activities of Daily Living; IADL: Instrumental Activities of Daily Living

### MitoChip v2.0 resequencing array protocol and array analysis

Mitochondrial DNA sequences were from a previous study [49] in which DNA was processed and analyzed as follows. Total DNA from whole blood of AD patients and age-matched controls was extracted by QIAamp DNA Blood Mini kit (Qiagen, Hilden, Germany) and amplified by REPLI-g mitochondrial DNA kit (Qiagen, Hilden, Germany). Purified DNA was fragmented and labeled by Genechip Resequencing array kit (Affymetrix, Santa Clara, CA). MitoChip arrays were loaded with DNA, then washed and stained (Fluidics Station 450) before being scanned in Affymetrix GeneChip Scanner 3000 7G.

Affymetrix MitoChip v2.0, allowing mtDNA sequences analysis with high reproducibility and sensitivity [50,51], is a mtDNA sequencing array with eight 25-mer probes/base position (four oligonucleotide probes/strand) corresponding to the whole revised Cambridge Reference Sequence (rCRS). Each 25-mer probe is varied at the central position to incorporate each possible nucleotide (A, G, C, or T). Data set were acquired by the Affymetrix Genechip Command Console (AGCC) software and analyzed with GSEQ 4.1. This software elaborates fluorescence intensity data by means of an algorithm whose parameters were defined to achieve optimal performance in analyzing mitochondrial sequences, with “model type” set at diploid to enable the detection of heteroplasmy and “quality score threshold” set at 3 to provide the best base calling accuracy and rate. We included in the analysis chips whose call rate was >95% implying that unclassified nucleotide positions (nps), known as no-calls, had to represent a very small percentage of total calls.

The output files used for our analysis were the Single Nucleotide Polymorphism (SNP) View files that provide, for each np, the corresponding base call. If the call is different from the corresponding rCRS base, we are in the presence of a mutation which is classified by the software as homoplasmic or heteroplasmic. Data discussed here were deposited in NCBI’s Gene Expression Omnibus [52] and are accessible through GEO Series accession number GSE49160 (www.ncbi.nlm.nih.gov/geo/query/acc.cgi?acc=GSE49160).

**Figure 1.**
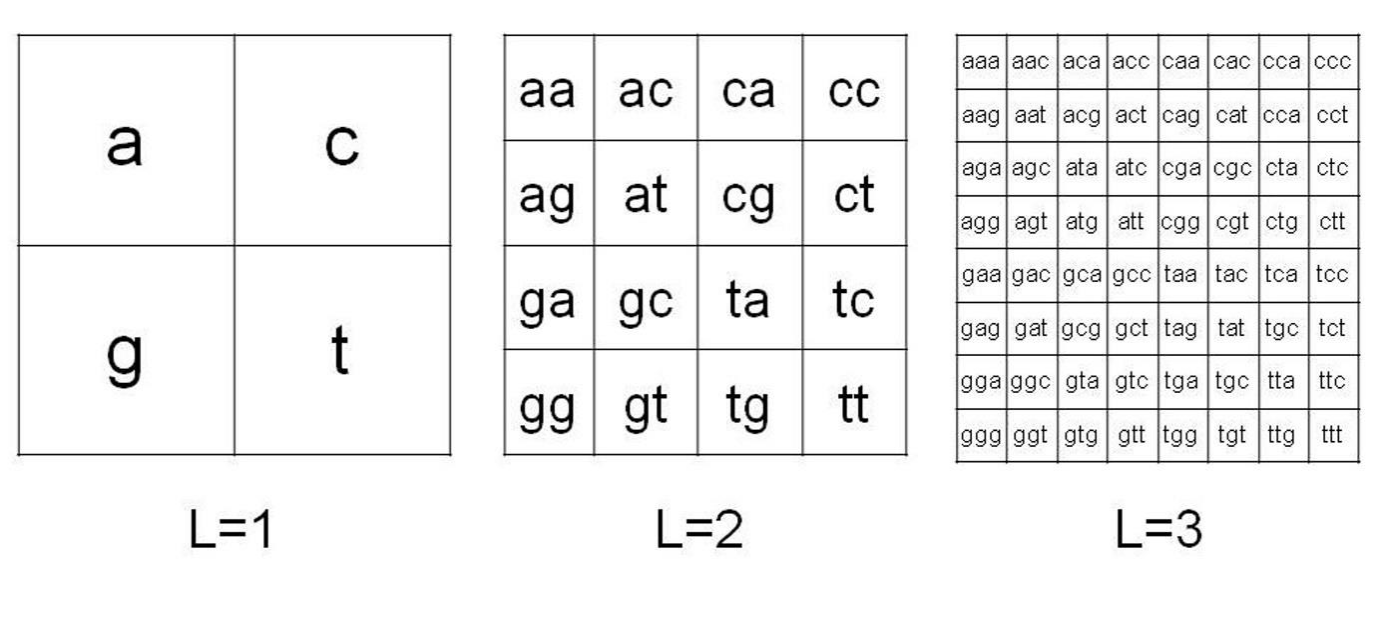
**Chaos Game Representation method**. CGR organization for *L*=1,2,3 in the case of mtDNA four-symbol alphabet sequence.

### Chaos Game Representation of mtDNA

Data set of mtDNA from Affymetrix system were transferred into a personal computer to be processed and analyzed as follows. The Chaos Game Representation (CGR) method was used to analyze the structure of mtDNA, see Jeffrey [41] for the pioneering work on this subject. This is a method that produces images from long sequences; therefore, it can be also applied to DNA information. An important problem in CGR theory is the connection of the genetic properties of DNA sequence and the fractal features of the corresponding CGR images. Let *S* be a DNA sequence; it can be seen as a finite string with respect to the alphabet {a, c, g, t}. The CGR image of *S* describes the frequency occurrence of all the possible substrings with fixed length *L*. Due to the four-symbol alphabet of *S*, this map can be organized in a square matrix of order 2*^L^*. Figure 1 shows a pictorial description of this organization for *L*=*1,2,3*.

A precise definition of this organization can be given by matrix tensor product; in fact, defining *M_1_* as the 2x2 matrix in the case *L*=*1*, matrix *M_L_* in the generic case *L* is defined as follows:

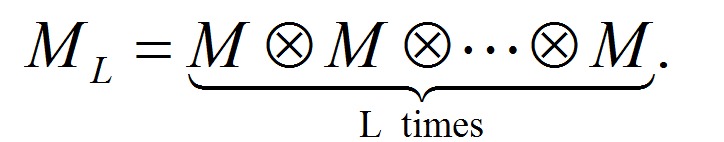


From this definition, for every integer *L*, a well-defined position *p(s)* is associated to each possible substring *sϵS* of length *L*.

Thus, given a positive integer *L*, the CGR matrix *A* of a given DNA sequence *S* can be constructed by using a simple algorithm:

1. initialize *A* to a zero matrix having *2^L^* rows and *2^L^* columns,
2. for each substring *sϵS* increase by one the entry of *A* having position *p(s)*.

An efficient implementation of this algorithm in a computer code is not a trivial task, see Vinga et al. [53] for details.

We propose a slight modification of the original algorithm in order to deal with undetermined DNA typing symbols (heteroplasmic mutations). In particular, substrings s having undetermined symbols are considered as the multiple strings generated by solving these undetermined symbols; each one of such strings having a fractional weight depending on the number of generated strings.

For the sake of clarity an example (*L*=*5*) is reported. String *s*=’*tamcg*’, where ‘*m*’ means ‘*a*’ or ‘*c*’, is expanded in *s_1_*=’*taacg*’ and *s_2_*=’*taccg*’ having weigth 1/2. String *s*=’*tavcg*’, where ‘*v*’ means ‘*a*’, ‘*c*’, or ‘*g*’ is expanded in *s_1_*=’*taacg*’, *s_2_*=’*taccg*’, *s_3_*=’*tagcg*’ having weight 1/3.

In this way, the above mentioned algorithm can be easily adapted to work also with substrings containing undetermined symbols; in this case, substrings containing a heteroplasmic mutation are expanded in multiple strings that are processed by the above algorithm with the exception that the string weight is used for the increment of CGR matrix entry.

The algorithm above described for CGR was implemented in software such that, while generating a matrix, it also provides a report with additional information on mtDNA sequence processed, i.e. type and number of nucleotide(s), number and position of homoplasmic/heteroplasmic mutations.

### Estimate of lacunarity

Fractal lacunarity analysis of mtDNA images as generated by CGR was performed by adopting a method previously developed in our laboratory as described in [45,46] and modified in [47,48].

In particular, among the definitions and calculating procedures proposed to estimate lacunarity, we chose the gliding box algorithm, GBA, based on the analysis of mass distribution in the set [40]. This method involves the variance of the box mass, *M*, at each step, wherein the box is moved one space unit at a time. The box mass is recounted till the whole region is traversed thus producing a frequency distribution of box masses, *n(M,b)*, where *b* is the size of the gliding box.

For the sake of simplicity we assume that, for each *b*, only a finite number of masses *Mj*, *j=1,2,,μ(b)* are encountered in the various gliding boxes of size b; therefore, a discrete frequency distribution *n(Mj,b), j*=*1,2,, μ(b)* has to be considered. Note that such an assumption holds for binary images, where the mass of a generic box on the image is given by the number of white pixels in the box, i.e. the pixels associated to the value one. From standard arguments on probability the moments of order *q* of *M*, are given by:

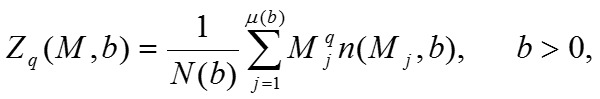
where the division by *N(b)*, i.e. the total number of boxes, needs to convert *n(Mj,b), j*=*1,2,,μ(b)* into a probability distribution. The definition of lacunarity function Λ uses only the first and the second moments of *M*, that is

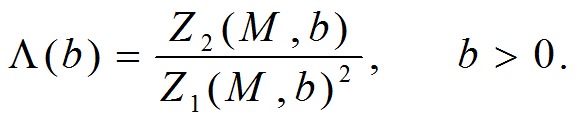
A simple extension of this algorithm can be used to deal with gray scale images. In this extension the moment formula for the discrete distribution of *M* is upgraded with the moment formula for *M* taking a continuous range of possible values, see Zaia et al. [47] for details. The efficiency of such an extension is usually improved by a different pre-processing step through a sigmoid function. In particular, this new pre-processing step prescribes to consider image *J*, in place of image *I*, where the pixels are defined as follows:

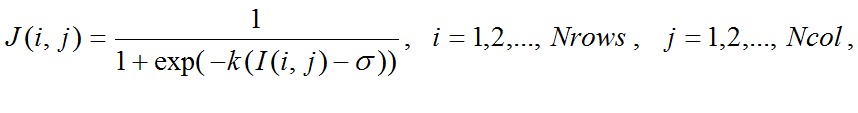
and *k*, *σ* > *0* are two given parameters. It is worth noting that the procedure goes toward a complete binarization by increasing parameter *k*, related to sigmoid regularization. The GBA method was implemented in software using MATLAB software package (the MatWorks, Inc.). The program begins to elaborate a gray-scale image coming from the above mentioned manipulation of mtDNA images.

It calculates the values of lacunarity, for each integer value of *b* between *b_min_* and *b_max_*, where *b_min_*, *b_max_* are given integer multiples of the pixel size in the image under consideration. Once the lacunarity function Λ*(b), b*=*b_min_, b_min_+1,, b_max_* is obtained, the program shows the results on a graph.

As expected from the asymptotic properties of function Λ on fractal set [40], for all the images analyzed, the behaviour of lacunarity function was a curvilinear plot resembling the hyperbola one; therefore, the following model:

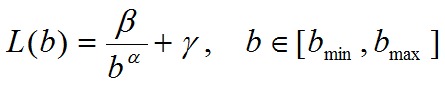
was chosen to approximate the lacunarity function [45]; note that *α*, *β*, *γ* are suitable parameters.

This observation is consistent with the theoretical behaviour of lacunarity function Λ for self-similar fractals and for other different random sets. Moreover, for such fractals, parameter *α* is related to DF of the set and parameter *β* characterizes the lacunarity of the set [45,46]. In each particular example considered in this study, the best interpretation of lacunarity Λ*(b)*, *b*=*b_min_, b_min_+1,, b_max_*, by the model function *L(b)*, *b*? [*b_min_*, *b_max_*], was computed as the solution of a least squares problem, where parameters *α*, *β*, *γ* are the independent variables. In particular, the minimizer of this problem is a triplet of parameters (α*, β*, γ*) of the model function that better represents the variation of mass density of pixels in that image.

### Statistical analysis

All data with normal distribution were presented as means ± SD. Student’s *t*-test was used to compare differences between AD and control groups. Statistical significance was accepted for *p* ≤ 0.05.

**Figure 2.**
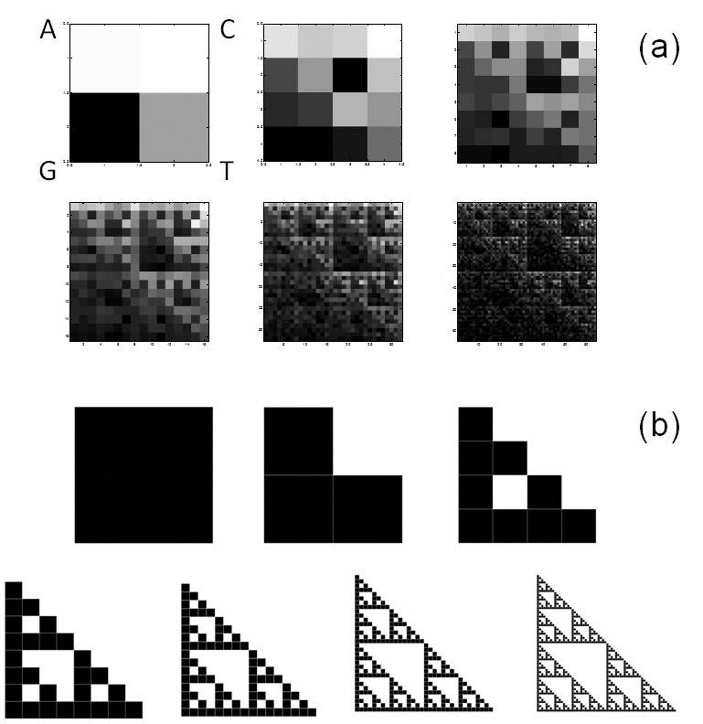
**Chaos Game Representation of human mtDNA**. Whole revised Cambridge Reference Sequence processed by CGR method generates matrices 2*^L^*x2*^L^*. Matrices for *L*=1 to *L*=6 are reported (a). CGR representation of human mtDNA resembles self-similarity of the triangle of Sierpinski, an ideal fractal built through repeated iterations starting from a square (b).

## RESULTS

Our method of fractal lacunarity analysis based on hyperbola model function was systematically applied to CGR images generated by rCRS, mtDNA sequences from 5 AD patients, and mtDNA sequences from 5 age-matched controls to set up the method and test its robustness. In particular, for any set of matrices 2*^L^*x2*^L^* (with *L*=1,2,….8) generated with CGR method, we obtained an identical set of reports for each mtDNA sequence processed for six times (data not shown). We also observed that for frame length L>8 a clear pictorial representation of the whole mtDNA fractal structure decreased because an increasing number of positions in the matrix are empty as the related substrings are rare or absent. This is consistent with the limited range of scale in which fractal properties can be observed in biological objects [2,35,39].

Twenty-eight mtDNA sequences from 14 AD patients and 14 controls were processed and analyzed to verify the potential of our lacunarity parameter *β* in characterizing alterations of mtDNA in aging and Alzheimer’s disease.

**Table 2:** Characteristics of mtDNA sequences processed by the proposed method

Number	rCRS	AD patients	Controls	*p* Value
Subjects		14	14	
Adenosine	5117	5178.8 ± 9.4	5174.9 ± 7.4	0.115
Cytosine	5175	4937.3 ± 28.9	4948.6 ± 11.9	0.095
Guanine	2163	2266.1 ± 15.0	2264.3 ± 13.6	0.371
Thymine	4089	4161.9 ± 12.4	4156.3 ± 15.0	0.076
Total mutations		18 ± 10	23 ± 12	0.094
Homoplasmic		14 ± 8	18 ± 9	0.090
Heteroplasmic		3 ± 3	5 ± 4	0.119

Values are expressed as mean ± SD;

*p* Values have been calculated by one-tail t-test to compare differences between AD and Controls groups;

rCRS: revised Cambridge Reference Sequence; AD: Alzheimer’s Disease

### Chaos Game Representation of human mtDNA

Figure 2 shows a set of six CGR images generated from rCRS with matrices for *L*=1 to *L*=6. Note that fractal structure of human mtDNA starts to be evident for *L*=3. It resembles the Sierpinski triangle (Figure 2b). This observation is consistent with Wang et al. [54] that reported the same Sierpinski-like structure for human mtDNA, different from human nuclear DNA and from DNA of other species thus highlighting a species- and type-specificity of DNA fractal representation.

**Figure 3.**
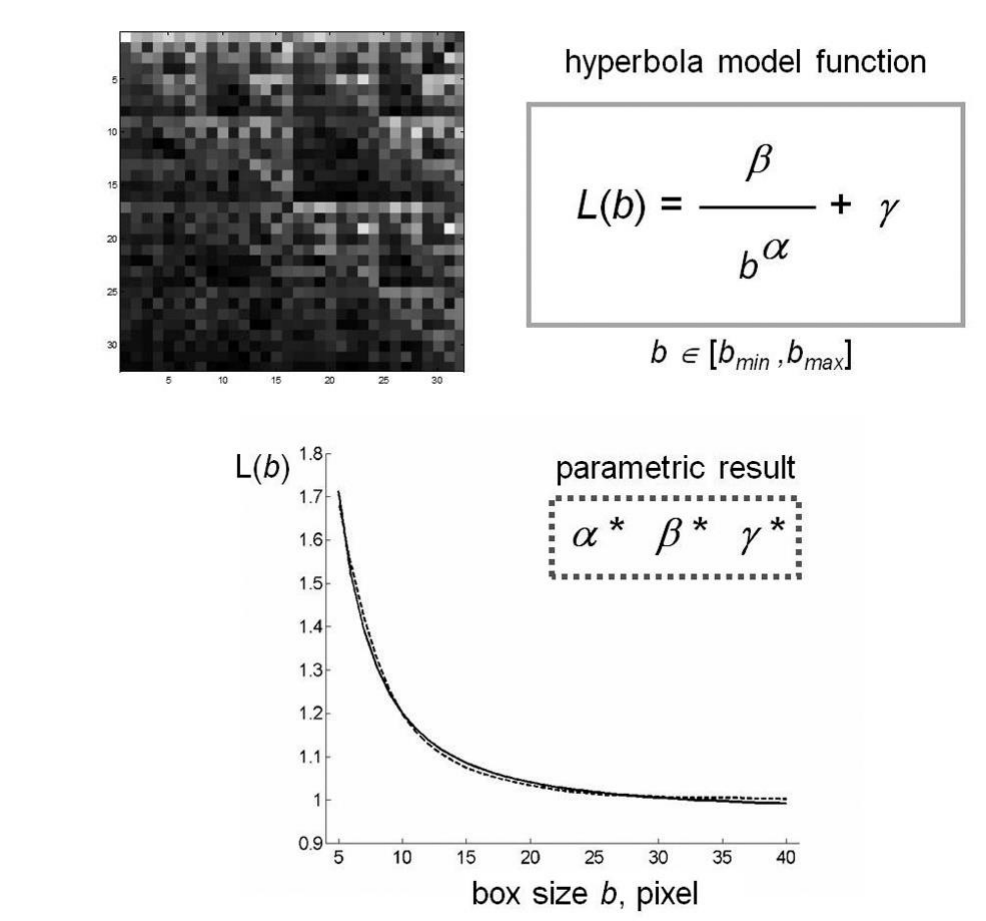
**Schematic representation of fractal lacunarity analysis**. (Top left) rCRS mtDNA image generated by CGR matrix for *L*=5 is a 32x32 square. The plot (bottom) represents the result of GBA application (dotted line), for *b_min_*=3, as fitted by hyperbola function (solid line) used to calculate the triplet of parameters *a, b, γ*. rCRS: revised Cambridge Reference Sequence; Chaos Game Representation; GBA: Gliding Box Algorithm.

### Fractal analysis of mtDNA in aging and Alzheimer’s disease

Our method of fractal lacunarity analysis was systematically applied to CGR images generated from the above described mtDNA sequences for *L*=4 to *L*=7 to find the proper combination of coefficients for the best characterization of any mtDNA sequence analyzed. Both *b_min_* and sigmoid coefficients (*k* and *σ*) were considered: we found that for *k* equal to 7 and *σ* equal to 0.7 we obtained comparable results for both *b_min_* equal to 3 and 5 when applied to matrices 2*^L^*x2*^L^* for *L* equal to 5 and 6.

Figure 3 shows a schematic representation of the method applied to a CGR matrix generated by rCRS. CGR matrices generated from all the mtDNA sequences produced a similar curvilinear plot. The almost perfect overlap of the two experimental and theoretic curves supports the appropriateness of our choice of hyperbola model function to fit the gliding box curve.

**Figure 4.**
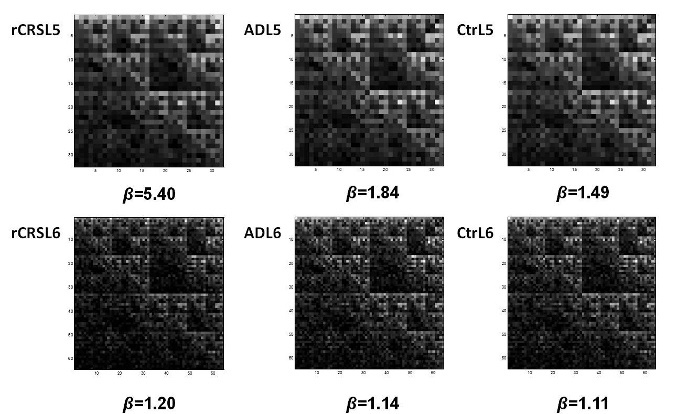
**Examples of CGR images of mtDNA sequences from different subjects**. Matrices for *L*=5 (top) and *L*=6 (bottom) generated from mtDNA of rCRS (left), AD patient (middle), and Control (Ctr, right). Lacunarity parameter *b* value for each representation is reported. CGR: Chaos Game Representation; rCRS: revised Cambridge Reference Sequence; AD: Alzheimer’s Disease.

Examples of mtDNA CGR images for *L*=5 and *L*=6 from rCRS, AD patient, and age-matched control are reported in Fig. 4. In spite of a similar display among the three kind of mtDNA sequences, parameter *b* values significantly differ for both frame lengths considered. In particular, lower *b* values, observed in AD and Control mtDNA when compared to rCRS, correspond to a higher degree of mutations of the nucleotide sequences considered.

Results in Table 3 were obtained from the application of our lacunarity method by using *b_min_* equal to 3 pixels to CGR images from matrices with *L* equal to 5 generated from mtDNA sequences of 14 AD patients and 14 Controls.

It is worth noting that fractal lacunarity parameter *β*, representative of mutation-related changes of the sequence, is significantly lower in both AD and Controls subjects when compared to rCRS. In addition, a statistically significant difference exits between AD and Control mtDNA with a higher degree of mutated frames in Controls (lower *β* values).

### Correlation of parameter β with classic diagnostic indices of Alzheimer’s disease

**Figure 5.**
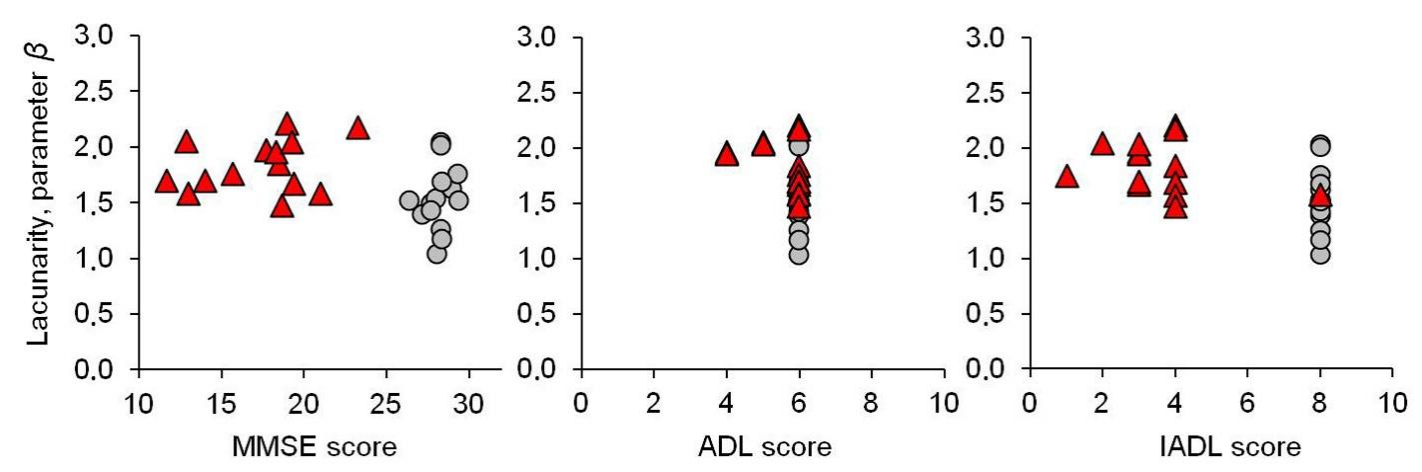
**Correlation of lacunarity parameter *β* with classical diagnostic indices of Alzheimer’s disease (AD)**. MMSE-Mini Mental State Examination (left); ADL-Activities of Daily Living (middle); IADL-Instrumental Activities of Daily Living (right). Triangles and circles represent AD patients and age-matched controls respectively.

In spite of the small sample under study, in the attempt to assign a diagnostic power to our lacunarity parameter *β*, a comparison with classic diagnostic indices was performed (Fig. 5). A cut-off value of parameter *β* equal to 1.68 (median value from the whole sample) identifies 71% of AD patients (10 out of 14, true positive) and 29% of Controls (4 out of 14, false positive) against a 100% of AD by MMSE, 93% by IADL tests, and 29% by ADL test. Cognitive and neuropsychological assessment, based on MMSE, ADL, and IADL tests, represents the first choice in clinical practice to diagnose AD. Other diagnostic approaches, described in Table 4, follow to complete the clinical setting in doubt cases.

## DISCUSSION

One most relevant aspect of aging populations is represented by the high degree of inter-individual variability. Ever increasing number of people is given to live longer; however, only a little percentage ages well. More than 60% of elderly people experience a pathologic aging, often characterized by co-morbidity. Heterogeneity of senescent phenotype has been, for long time, considered as an obstacle to understand and recognize “physiologic” aging, therefore making hard discriminating pathologic aging subjects. This is what is happening also in the case of Alzheimer’s disease.

Here we show that fractal analysis represents a powerful tool to discriminate between aging and age-related pathologies. In particular, fractal lacunarity analysis of mtDNA, performed by the proposed method, is able to highlight a statistical significant difference of mtDNA mutations phenotype between AD patients and age-matched controls, thus overcoming the limits of classical numerical and statistical methods.

From the literature, it is quite accepted that mitochondrial dysfunction and oxidative damage play an important role in the pathogenesis of AD [55-60]; however, the mechanisms involved have not been clearly identified yet [61-64]. Several studies since 90s have failed to find consistent mutational abnormalities in AD mtDNA beyond those associated with aging, with most studies carried out in postmortem brain. As a matter of fact, sporadic mutations have been found increased in mtDNA control regions in AD patients compared to controls [65]; however, a study on Japanese patients failed to find any causal role of mtDNA mutations in the etiology of the disease [66]. It has been also reported that pathologic inherited mtDNA mutations do not represent a major etiological factor in sporadic AD; however, it seems that at least a little percentage of AD patients carry pathologic mtDNA mutations and a little percentage of normal aged individuals carry mtDNA mutations that protect them from AD onset [67]. These changes have been found both in brain and peripheral tissues, thus suggesting that AD can be regarded as a systemic disease [68].

**Table 4:** Different approaches to AD diagnosis used in clinical practice

	Target	Advantages	Disadvantages
Cognitive and neuropsychological assessment	Provides a detailed picture of cognitive status. Thinking skills that are explored include memory, language, visual-spatial perception, attention, motor function, and executive function (e. g. MMSE, NPI, ADL, IADL)	Identifies very early subtle cognitive changes and which areas of mental functioning are affected. It can help distinguish AD from other forms of dementia. The cost is low and the tests are not invasive.	An abnormal result can have many explanations other than AD. It can miss cognitive impairment in those who are highly educated. It can be tiring and stressful for patients being tested.
Brain imaging	CT scans and MRI examine structural changes of the brain. PET scans can show metabolic changes and amyloid deposition.	Allows finding possible other causes of dementia symptoms (brain trauma, tumor, or stroke). PET scans can help distinguish AD from frontotemporal dementia.	Brain imaging may require the use of intravenous "tracing" agents, that can cause side effects. MRI scanners can induce claustrophobia and may not be compatible with pacemakers or other devices. The cost is notably high.
Spinal tests	The amounts of three AD biomarkers, amyloid-β 42, total tau, phosphorylated tau, are determined in CSF through a lumbar spine puncture.	CSF biomarkers can identify patients without clinical or preclinical signs of AD. A low level of amyloid-β 42 in patients with mild cognitive impairment seems to predict with 80-90 % accuracy who will not develop AD.	It is an invasive test to be performed by an expert high qualified specialist. Risk exists for infection, ble eding, and pain .

MMSE: Mini-Mental State Examination; NPI: Neuropsychiatric Inventory; ADL: Activities Daily Living; IADL: Instrumental Activities of Daily Living; AD: Alzheimer Disease; CT: Computed Tomography; MRI: Magnetic Resonance Imaging; PET: Positron Emission Tomography; CSF: Cerebrospinal Fluid

Point mutations do not necessarily lead to the development of disease(s). The mitochondrial genome shows a high degree of polymorphism because of mutations, and mitochondria within cells differ from each other depending on their mitochondrial genome profile (heteroplasmy). However, significant differences in biochemical phenotype and pathological impact do not occur until the number of mutated mtDNA molecules reaches a critical threshold level of bioenergetic impairment which depends on both the type of accumulated mutations and the specific ATP requirement of the tissue [69].

According to “mitochondrial cascade hypothesis” two main events are responsible of disease onset: the rate of mitochondrial decline determined by inherited and acquired mutations, and the point at which adequate compensation and adaptation to age-related changes are no longer available. The kinetics of these two events define the age at which patients begin to experience clinical signs of the disease [70]. Severity of the symptoms has been correlated with the degree of heteroplasmy and the size of possible deletions [71]. The degree and pattern of neurodegeneration within patients with mitochondrial disease can vary depending on the type of mutations and mutation load segregation at level of brain regions as a whole as well as at level of single neurons [17].

In this context, it sounds appropriate to call in cause the theory of complexity and the laws of chaos to explain and characterize individual phenotypes evolving over time as ‘normal’ aging or pathologic aging [2]. As above introduced, the variability observed in senescent phenotype can be explained in the light of the theory of complexity by considering longevity as a “secondary product” of evolution of a dynamic nonlinear system. In fact, biological systems, human beings in particular, can be considered complex systems, made up of numerous sub-systems (nervous, cardiovascular, endocrine, … systems), interacting with each other. Each sub-system is further subdivided into interacting lower components (organs, tissues, …) and so on at lower levels of organization. In particular, the organization into hierarchy and the laws of chaos can be called in cause to explain their evolution, from development to senescence through the maintenance of “homeostatic” dynamic equilibrium of all their integrated functions as an adaptive response to continuous noxae from both endogenous and exogenous environments.

In the light of this holistic point of view, aging has been defined as the temporal evolution of a complex system, characterized by a nonlinear dynamic behavior governed by the laws of chaos. The system evolves, under the influence of both endogenous and exogenous environments, with loss of complexity during aging [6,7,19].

Human beings, as complex systems with a chaotic behavior, generate fractals: they can be observed at both structural and functional levels. Therefore, fractal analysis emerges as a suitable tool to measure biocomplexity and its changes with aging and pathology. Fractal analysis can measure variations of complexity in biosystems that, evolving with time, follow different trajectories. The senescent phenotype evolves as ‘normal’ aging, pathologic aging, or successful aging depending on the individual specific genetic-environment interactions [1,2].

As mentioned in the introduction, dealing with mtDNA mutations as gaps in the nucleotidic sequence, fractal lacunarity represents a suitable tool to differentiate between aging and AD. Fractal lacunarity, in fact, provides a holistic estimate of changes in mtDNA sequences comprising of number, type, and dislocation of mutation(s), the combination of which probably contributes to AD onset and progression.

Lacunarity analysis of genomic sequences has been recently proposed as a potential bio-sequence analysis method in both prokaryotes and eukaryotes [72]. Our original method, based on hyperbola model function to quantify fractal lacunarity, was previously successfully applied to trabecular bone images to discriminate between aging and osteoporosis [45,46]. Here we propose a modified version [47,48] reviewed and adapted to operate on CGR images of mtDNA sequences to characterize individual mutation profile of mitochondrial genome. It is worth noting that mtDNA accounts for both homoplasmic and heteroplasmic mutations, the last ones identified in GSEQ 4.1 software by symbols other than four-symbols alphabet commonly used to represent DNA sequences. As far as in our knowledge, the modification of the original CGR algorithm introduced in this study to deal with heteroplasmic mtDNA mutations (undetermined DNA typing symbols) represents a novelty in the field. Parameter *β* from our original bio-mathematical model, representative of lacunarity, is sensitive to mtDNA sequence changes and is able to discriminate between AD patients, with clinical signs of disease, and age-matched controls. Lacunarity *β* value cut-off at 1.68, median value from the whole sample, identifies most AD patients. It is worth noting that the gold standard for AD diagnosis is represented by histological identification of amyloid plaques that can be performed only at the autopsy. All AD diagnostic tools used in clinical practice have some drawbacks and none of them can recognize AD with 100% accuracy and specificity. Therefore, the identification of new AD biomarkers would be very useful, though the physio-pathological mechanisms of AD onset remains unknown. Phenotyping mtDNA mutations in AD by the proposed method could provide a clear-cut support to the hypothesis on AD etiopathogenesis based on a cause-effect relationship between accumulation of mtDNA mutations and AD-related neurodegenerative changes. Particularly intriguing is the presence of a little percentage of controls showing a mtDNA mutation profile similar to AD: they could represent the lecture key to identify mtDNA mutation(s) that protect aged individuals from AD onset as well as pathologic mtDNA mutation(s) that predispose to the disease. Geneticists could use the proposed method as an easy and fast genomic screening tool to recognize altered nucleotidic sequences and then focus on specific mutation(s) found in particular nucleotide frame(s) of interest for onset and progression of AD. It has to be stressed that the proposed method has potential for a wide application to any pathology with genetic implications.

In conclusion, fractal lacunarity analysis represents a useful tool to analyze mtDNA mutations. Parameter *β* from our method is able to characterize individual mutation profile of mitochondrial genome and appears a promising index to discriminate between AD and aging.

Results presented in this paper are from a pilot study to set up the method and need to be confirmed in larger samples of patients in which gender- or even race-related differences could be also investigated. Anyhow, these results give an interesting perspective: the quantitative characterization of mutated mtDNA features by fractal analysis. In this direction, the present study deserves further developments by exploring the other fractal properties, DF and succolarity, as well as other texture measures such as statistical approaches, co-occurrence matrix descriptors, power spectrum analysis. Exploiting the synergy among fractal measures to improve both texture recognition [73] and complexity characterization of mtDNA could make the difference in discriminating AD patients and controls having the same lacunarity parameter *β*.

Improvements of the method are in progress for a morewide application as a smart screening tool to recognize altered long biological sequences (genomic and proteomic). It could represent a promising approach to give insight into the search of biomarkers of aging and pathologies.
